# The European War Fund Formed by the St. John Ambulance Department

**Published:** 1914-12-19

**Authors:** Agnes Jekyll

**Affiliations:** St. John's Gate, Clerkenwell, London, E.C.


### The European War Fund Formed by the St. John Ambulance Department.

*To the Editor of* The Hospital.

Sir,—The Ambulance Department of the Order of St. John of Jerusalem appeal to the generosity of a most generous public for renewed support. The hospitals are counting on our help as we have come to count on that of our wonderful working parties and good friends, known and unknown, throughout the country. Daily the demands on us multiply and increase.

From all parts of Great Britain, from France and Belgium and Servia, from Indian camps and hospital ships, from ambulance trains and motor convoys, come the most urgent appeals for clothing, bedding, surgical dressings, hospital comforts, and for warm garments for convalescents. Our shelves, at first so full, are getting empty, and daily the committee grow more anxious lest they should have to disappoint those who look to them for continued help. We want everything that is warm and useful and clean; everything that a wounded man or a sick man needs, from the time that his war-worn uniform is taken from him in hospital until he is ready to face the world again as a fighting man—or a cripple. We cannot respond to these appeals, or, we might say, claims, unless everyone will help us according to their measure and recognise the responsibility of every non-combatant to give to the utmost to those who are fighting so gallantly for our homes and our liberties. The address for all contributions is The President, St. John Warehouse, 56 St. John Square, Clerkenwell, E.C.—Yours faithfully, Agnes Jekyll.

St. John's Gate,
Clerkenwell, London, E.C.

